# Fenofibrate induces human hepatoma Hep3B cells apoptosis and necroptosis through inhibition of thioesterase domain of fatty acid synthase

**DOI:** 10.1038/s41598-019-39778-y

**Published:** 2019-03-01

**Authors:** Bang-Jau You, Mann-Jen Hour, Li-Yun Chen, Shu-Ching Luo, Po-Hsiang Hsu, Hong-Zin Lee

**Affiliations:** 1 Department of Chinese Pharmaceutical Sciences and Chinese Medicine Resources, China Medical University, Taichung, Taiwan; 2 School of Pharmacy, China Medical University, Taichung, Taiwan

## Abstract

This study demonstrated that fenofibrate, a lipid-lowering drug, induced a significant time-dependent cytotoxicity of hepatoma Hep3B cells. Hep3B cells are significantly more sensitive to cell killing by fenofibrate than hepatoma HepG2, lung cancer CH27 and oral cancer HSC-3 cells. From the result of docking simulation, fenofibrate can bind excellently to the thioesterase domain of fatty acid synthase (FASN) binding site as orlistat, a FASN inhibitor, acts. The fenofibrate-induced cell cytotoxicity was protected by addition of palmitate, indicating that the cytotoxic effect of fenofibrate is due to starvation of Hep3B cells by inhibiting the formation of end product in the FASN reaction. Inhibition of lipid metabolism-related proteins expression, such as proteins containing thioesterase domain and fatty acid transport proteins, was involved in the fenofibrate-induced Hep3B cell death. Fenofibrate caused S and G2/M cell cycle arrest by inducing cyclin A/Cdk2 and reducing cyclin D1 and E protein levels in Hep3B cells. The anti-tumor roles of fenofibrate on Hep3B cells by inducing apoptosis and necroptosis were dependent on the expression of Bcl-2/caspase family members and RIP1/RIP3 proteins, respectively. These results suggest that fenofibrate has an anti-cancer effect in Hep3B cells and inhibition of lipid metabolism may be involved in fenofibrate-induced Hep3B cells apoptosis and necroptosis.

## Introduction

Fibric acid derivatives are effective lipid-lowering drugs. Chen *et al*. (2012) have demonstrated that a decrease of triglyceride accumulation induced by fenofibrate, a fibric acid derivative, resulted from increase of adipose triglyceride lipase expression and decrease of fatty acid synthase (FASN) level under high-glucose condition in myoblast cells^1^. In addition to being lipid-lowering agents, fibric acid derivatives were also found to have anti-cancer effects through inhibition of FASN activity^2^. Treatment of clofibrate, a fibric acid derivative, significantly induced a decrease of the protein expression of active FASN and an increase in the amounts of free fatty acids in breast cancer^2^. Recently, it was reported that fibric acids trigger apoptosis or necrosis in human hepatoma cell lines^3–7^. Fenofibrate can trigger cancer cell apoptosis through activation of NF-κB pathway, which is independent on PPARα expression^8^. Drukala *et al*. (2010) indicated that fenofibrate-mediated PPARα-dependent ROS accumulation is an important factor in inhibition of glioma cell motility^9^. Inhibition of the metastasis of CAL 27 cells by fenofibrate was also demonstrated to be associated with the inhibition of AMPK and NF-κB signaling pathway^10^. However, a little-known fact about the anti-cancer effects of fenofibrate in inhibiting FASN and the molecular mechanisms underlying the FASN inhibition relationship on cell death remain unclear.

FASN is an important enzyme in the *de novo* lipogenesis pathway and plays a central role in obesity, nonalcoholic fatty liver disease (NAFLD) and cancer cell development^11–13^. FASN has also been found to be highly expressed in a wide variety of human cancers, including liver cancer, whereas overexpression of FASN is associated with increasing tumor progression, poor prognosis and risk of death^14–16^. These observations indicate that FASN plays a critical role in tumor lipid metabolism, and FASN-catalyzed biosynthesis of fatty acid should be a good target for tumor therapy. Recently, inhibition of FASN has been considered as an attractive target for cancer treatment, including hepatocellular carcinoma^13,17,18^. However, there are still no effective FASN inhibitors for cancer treatment. Therefore, the discovery of novel FASN inhibitors will be highly expected to treat cancers. NAFLD is a wide variety of liver disease related with obesity and the metabolic syndrome, and has shown to be a risk factor for developing hepatocellular carcinoma^19^. According to government reports, liver cancer is the second leading cause of death in Taiwan in 2017. To examine whether fenofibrate, a lipid-lowering drug, could induce anti-cancer effects on liver cancer, human liver cancer cell lines Hep3B and HepG2 were used in this study.

Molecular docking is a well-established computational technique, which was used to determine the interaction of two molecules and the best orientation of ligand. Therefore, molecular docking approach is used to predicting the predominant binding mode of a ligand with a protein of known three-dimensional structure. Reduction of the activity of FASN has been found to be an essential event in the tumor growth inhibition, which can be considered to be a novel strategy for cancer treatment. The catalytic Ser2308-His2481-Asp2338 triad, the active site of thioesterase domain of FASN, plays a key role in the hydrolysis of the thioester bond that links phosphopantetheine of ACP (acyl carrier protein) to the fatty acyl group^20,21^. Orlistat, a FDA-approved drug for obesity, was reported to bind the thioesterase domain of FASN, which can inhibit tumor growth and induce tumor cell death^22–24^. It has also been demonstrated that orlistat docked into catalytic triad resulted in prevention of the delivery of fatty acid from ACP to Ser2308 of thioesterase domain^20,21,25^. In order to predict whether fenofibrate has the same inhibitory effect on FASN activity as orlistat, fenofibrate was docked with 2px6, the crystal structure of thioesterase domain-orlistat complex^26^, in this study. Based on the result of molecular docking, fenofibrate should be an inhibitor of FASN through binding on the thioesterase domain, which is a similar result of orlistat docked thioesterase of FASN as previously described^25,26^. It interests us to investigate whether fenofibrate inhibits cancer cell growth through inhibition of FASN activity.

## Results

### Molecular docking

**Figure 1:**
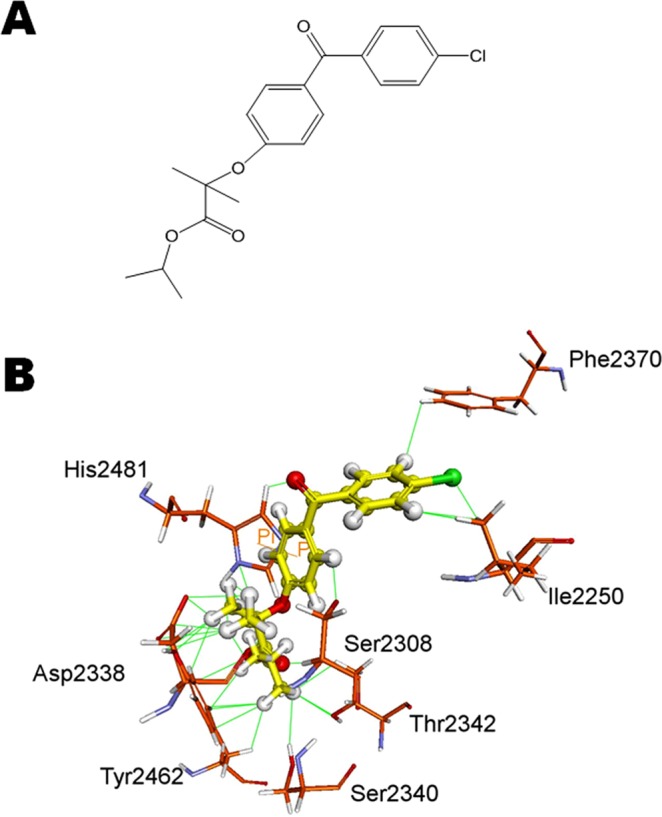
(**A**) Chemical structure of fenofibrate. (**B**) Molecular modeling. Fenofibrate (ball) docked well with active site of thioesterase of FASN. The interacting amino acids are shown as sticks and labelled. The interactions between fenofibrate and amino acid are shown as lines.

Fenofibrate (Fig. 1A) is known to have lipid-lowering effects, and it interests us to investigate whether fenofibrate inhibits cancer cell growth through inhibition of the FASN activity, similar to orlistat. In this study, fenofibrate was docked with 2px6, the crystal structure of thioesterase domain of FASN bound to orlistat. The result of fenofibrate docking into the thioesterase domain of FASN is shown in Fig. 1B. The interaction involved the Pi-Pi interaction between fenofibrate and His2481, and van der Waals interactions with Ile2250, Ser2308, Asp2338, Ser2340, Thr2342, Phe2370, Tyr2462 and His2481. These interactions allow fenofibrate to bind efficiently to the Ser2308-His2481-Asp2338 catalytic triad which is the active site of thioesterase domain of FASN. Furthermore, we found other key interacting residues, Ile2250, Thr2342 and Tyr2462, are also the same as hexanoyl tail of orlistat with thioesterase domain of FASN as described by Pemble *et al*.^26^ (Fig. 1B). In addition, the chlorophenyl group of fenofibrate may interact with Ile2250 and Phe2370 by van der Waals force (Fig. 1B). Based on the above reasons, we suggest that fenofibrate, like orlistat, binds to the thioesterase domain of FASN well, which could inhibit the activity of FASN.

### Effect of fenofibrate on cell death of Hep3B, HepG2, HSC-3 and CH27 cells

**Figure 2:**
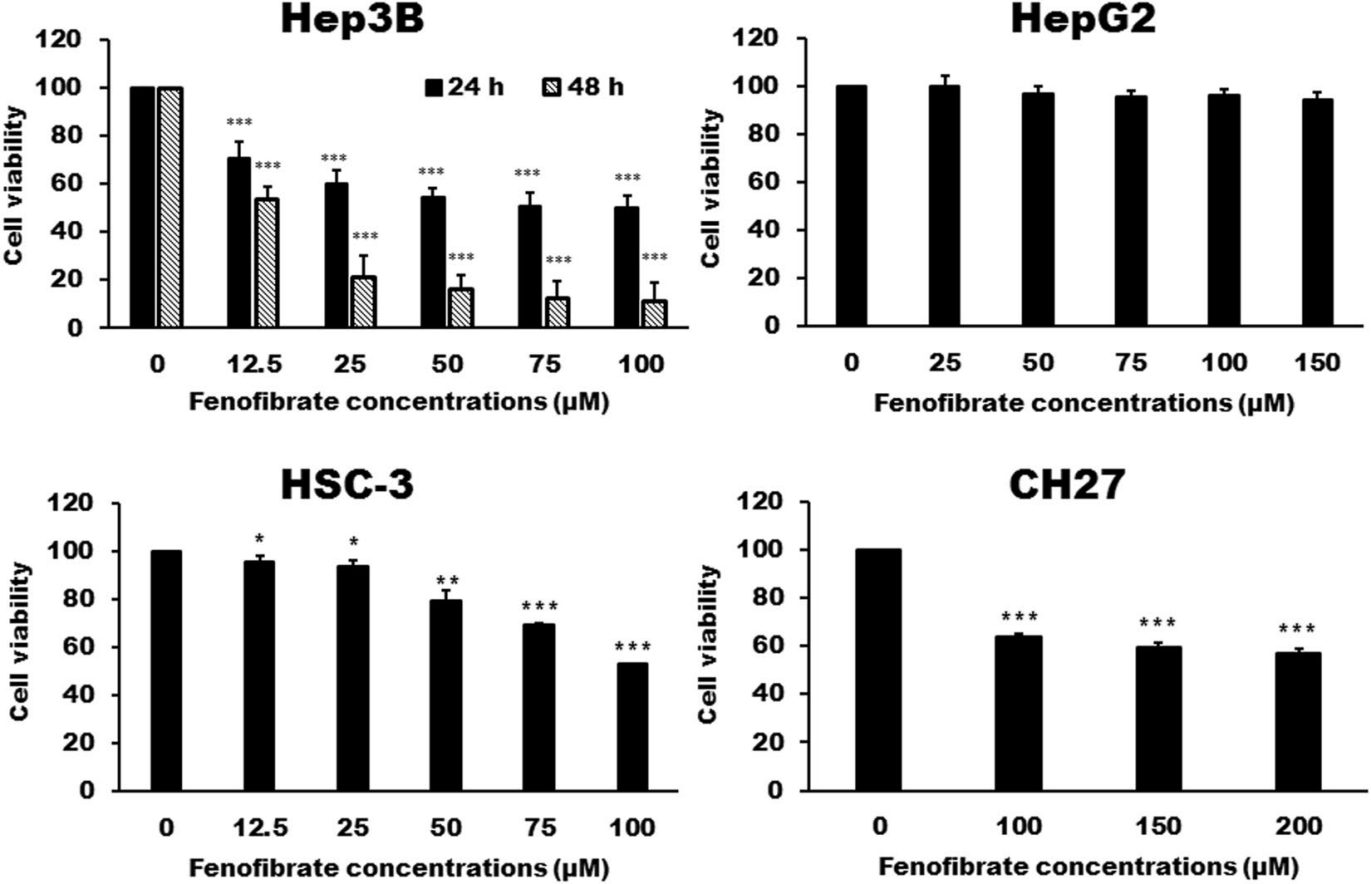
Cytotoxicity of fenofibrate in Hep3B, HepG2, HSC-3 and CH27 cells. Hep3B, HepG2, HSC-3 and CH27 cells were incubated with vehicle alone or with various concentrations of fenofibrate for 24 h. The cytotoxicity was assessed by Trypan blue exclusion assay and the viable cells were counted. The data are expressed as the mean percentage of control ± S.D. of four independent experiments performed in triplicate. ^*^P < 0.05, ^**^P < 0.01, ^***^P < 0.001 compared to the control values.

Based on the docking simulation results, we supposed that fenofibrate may be an inhibitor of FASN. Furthermore, the inhibition of FASN has been shown to have an anti-cancer effect in a wide variety of cancers and FASN was found to be highly expressed in liver cancer cells. Therefore, this study investigated the effect of fenofibrate on cell growth of liver cancer cell lines Hep3B and HepG2. As shown by Trypan blue exclusion assays, the treatment of Hep3B cells with fenofibrate for 24 or 48 h resulted in a significant cytotoxic effect (Fig. 2). These cytotoxic effects were time dependent but not dose dependent. Doses within the range of 50 to 100 μM had the same effects, about 50% Hep3B cell death (Fig. 2). However, fenofibrate had no significant cytotoxic effect on the HepG2 cells (Fig. 2). Experiments also demonstrated that fenofibrate had a cytotoxic effect on oral cancer HSC-3 and lung cancer CH27 cells (Fig. 2). However, Hep3B cells are significantly more sensitive to cell killing by fenofibrate than CH27 and HSC-3 cells. After 24 h of fenofibrate treatment, the concentrations of inducing 50% cell death by fenofibrate is more than 100 μM for HSC-3 cells and 200 μM for CH27 cells (Fig. 2).

### Effects of fenofibrate on the cell cycle distribution and the expression of cell cycle regulators in Hep3B cells

**Figure 3:**
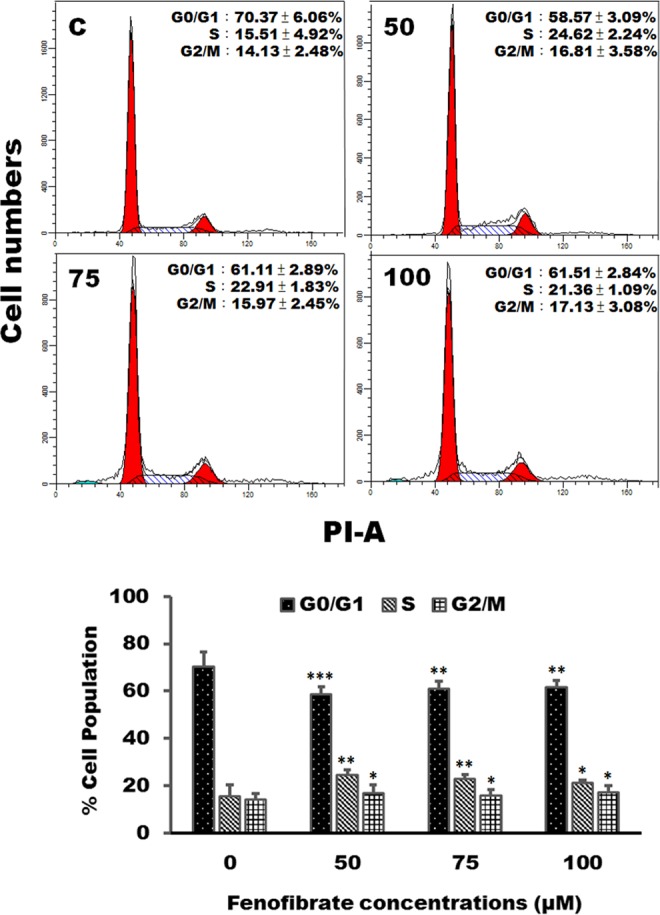
Fenofibrate induced cell cycle arrest in Hep3B cells. Cells were treated with vehicle alone or with 50, 75 or 100 µM fenofibrate for 24 h. After treatments, cells were collected and analyzed on a flow cytometer. Results are representative of four independent experiments performed in duplicate. ^*^P < 0.05, ^**^P < 0.01, ^***^P < 0.001 compared to the control values.

**Figure 4:**
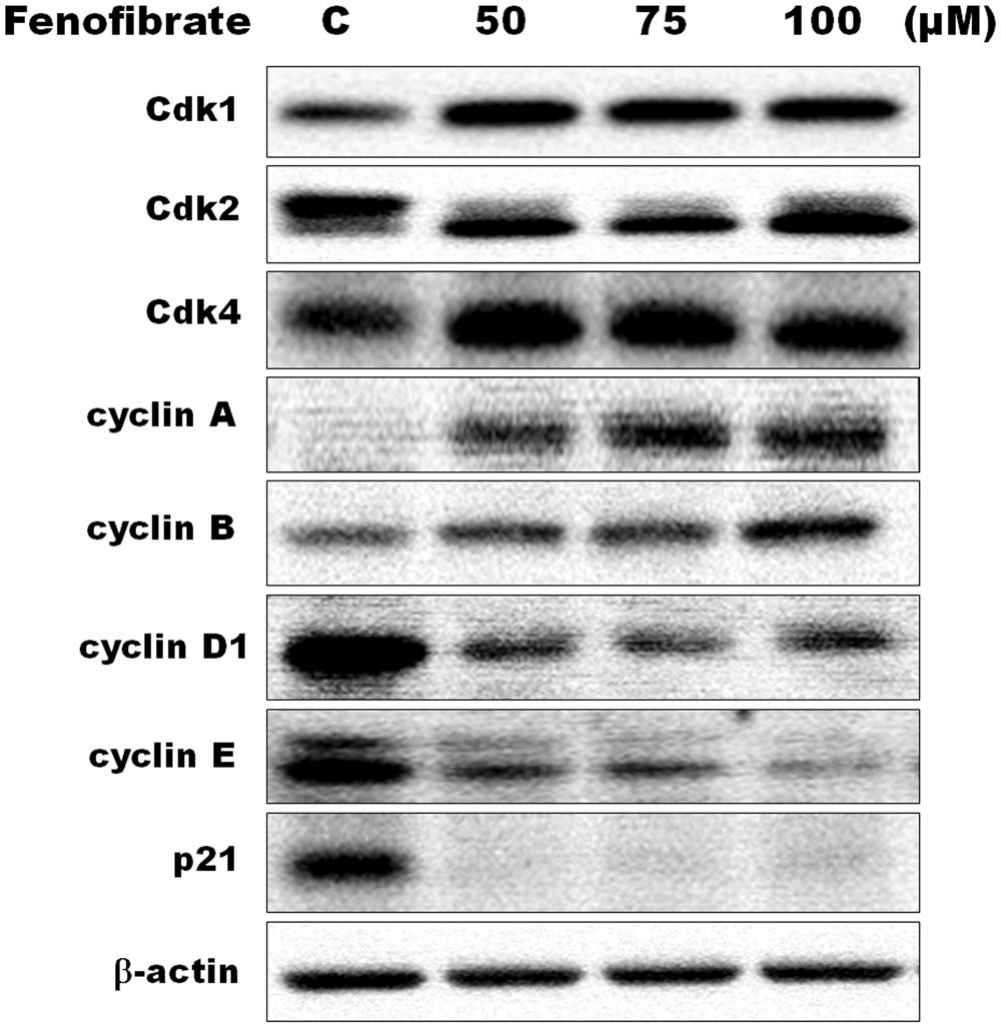
Effects of fenofibrate on the expression of cell cycle regulators in Hep3B cells. Hep3B cells were incubated with 0.1% DMSO or with 50, 75 or 100 µM fenofibrate for 24 h. Cell lysates were analyzed by SDS-PAGE (10% for cyclin A, cyclin B and cyclin E, 12% for β-actin, 13% for Cdk1, Cdk2, Cdk4 and cyclin D1 and 14% for p21), and then probed with primary antibodies followed by secondary antibodies. Results are representative of three independent experiments performed in triplicate. All of full-length gels and blots are included in Supplementary Fig. 4.

Since the fenofibrate triggered cell death of Hep3B cells, we investigated the effect of fenofibrate on the cell cycle distribution of Hep3B cells. After 24 h of vehicle treatment, the proportion of Hep3B cells in G0/G1 phase of cell cycle was about 70% (Fig. 3). In addition to S phase arrest, fenofibrate also leads to prolongation of the G2/M phase in Hep3B cells. Significant G2/M- and S-phase increase with a concomitant decrease in the number of cells in the G0/G1 phase was observed (Fig. 3). After Hep3B cells treatment with 50 µM fenofibrate, the increase in the percentage of cells in S phase was from vehicle treated 15.51 ± 4.92% to 24.62 ± 2.24% and the increase in G2/M phase was from vehicle treated 14.13 ± 2.48% to 16.81 ± 3.58%, with concomitant decrease in percentage of cells in G0/G_1_ phase from vehicle treated 70.37 ± 6.06% to 58.57 ± 3.09% (Fig. 3). Since fenofibrate caused G2/M and S phase arrest, the critical cell cycle regulators in the S and G2/M phase were examined after treatment with fenofibrate (50, 75 and 100 µM) for 24 h. As shown by immunoblotting, the decreases in cyclin D1 and E, G0/G1 phase progression regulators, protein levels were observed after treatment with fenofibrate (Fig. 4). Exposure of Hep3B cells to fenofibrate resulted in significant increases in cyclin A and cyclin B, which are involved in the regulation of S and G2/M phase progression, protein levels after 24 h treatment with fenofibrate (Fig. 4). It is noteworthy that two bands of Cdk2 appear at approximately 33 kDa. The signal of lower band of Cdk2 was found to gradually increase up to 100 μM of fenofibrate, whereas the upper band decreased (Fig. 4). The protein expression of Cdk1 and Cdk4 was increased during treatment with fenofibrate (Fig. 4). Fenofibrate also induced a significant decrease in the protein levels of p21 compared to the control cells in this study (Fig. 4).

### Fenofibrate-induced apoptosis of Hep3B cells was accompanied by necroptosis

**Figure 5:**
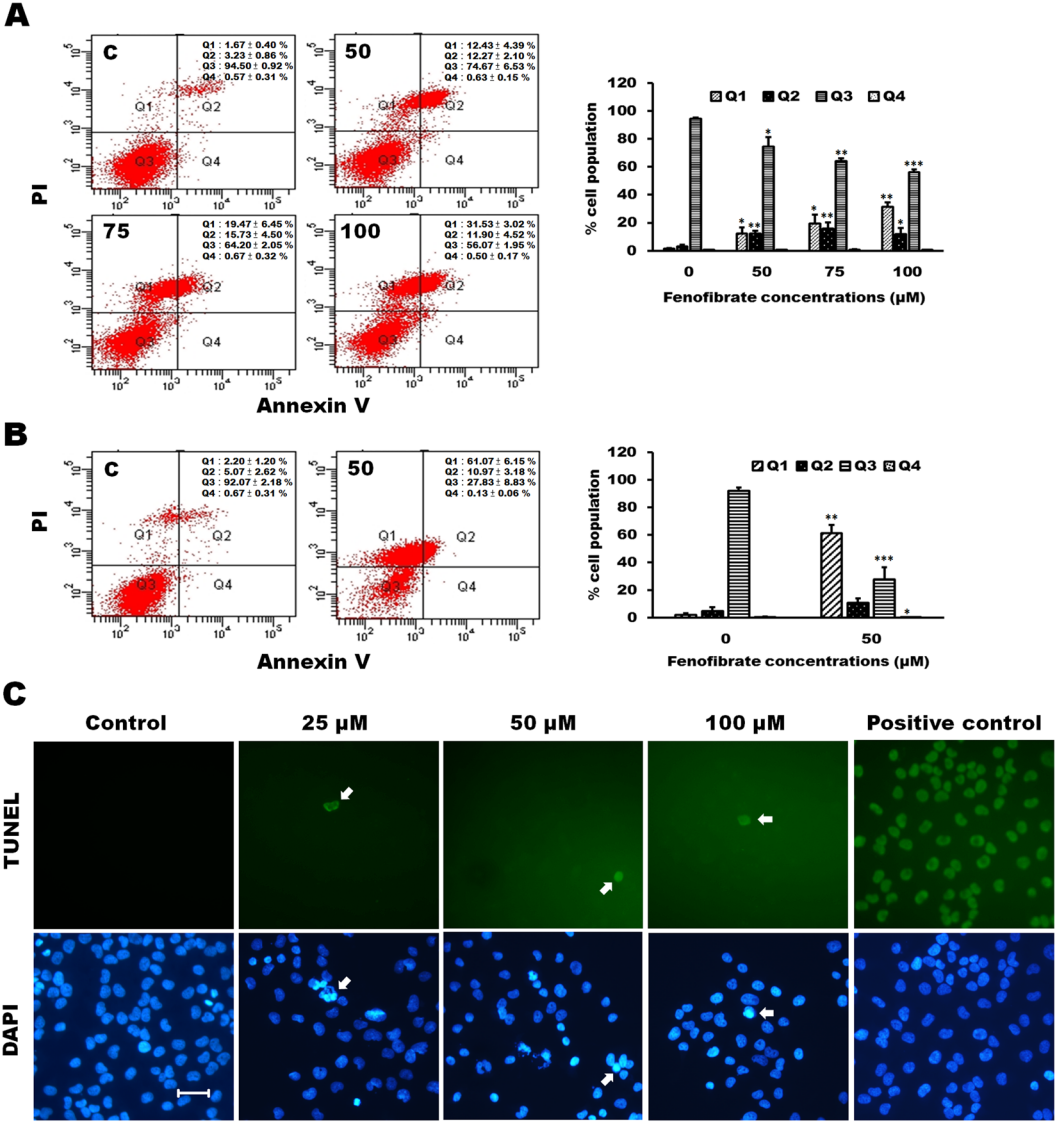
Fenofibrate induced apoptosis and necroptosis of Hep3B cells. (**A**) Cells were incubated with 0.1% DMSO or with 50, 75 or 100 µM fenofibrate for 24 h. Cells were then stained with annexin V-FITC/PI and analyzed using flow cytometry. The cell population of Q3 area was regarded as control cells, whereas Q4 area were taken as a measure of early apoptosis, Q2 area as late apoptosis and Q1 area as necrosis. All results are representative of three independent experiments performed in duplicate. ^*^P < 0.05, ^**^P < 0.01, ^***^P < 0.001 compared to the control values. (**B**) Cells were incubated with 0.1% DMSO or 50 µM fenofibrate for 48 h and then processed for annexin V-FITC/PI staining. All results are representative of three independent experiments performed in duplicate. ^*^P < 0.05, ^**^P < 0.01, ^***^P < 0.001 compared to the control values. (**C**) The effect of fenofibrate on the nuclear morphology and DNA fragmentation in Hep3B cells. The nuclear morphology and DNA fragmentation of fenofibrate-sensitized Hep3B cells were evaluated by DAPI and TUNEL assay. Hep3B cells were cultured for 24 h with vehicle alone or with 25, 50 or 100 µM fenofibrate. Cells were stained with TUNEL and then stained with 1 μg/ml DAPI for 5 min at 37 °C. In positive control, Hep3B cells were incubated with 200 U/ml DNase I for 10 min after cell permeabilization and then stained with TUNEL. After three washings in PBS, the cells were examined by fluorescent microscope. Results are representative of three independent experiments performed in triplicate. Bar = 25 μm.

**Figure 6:**
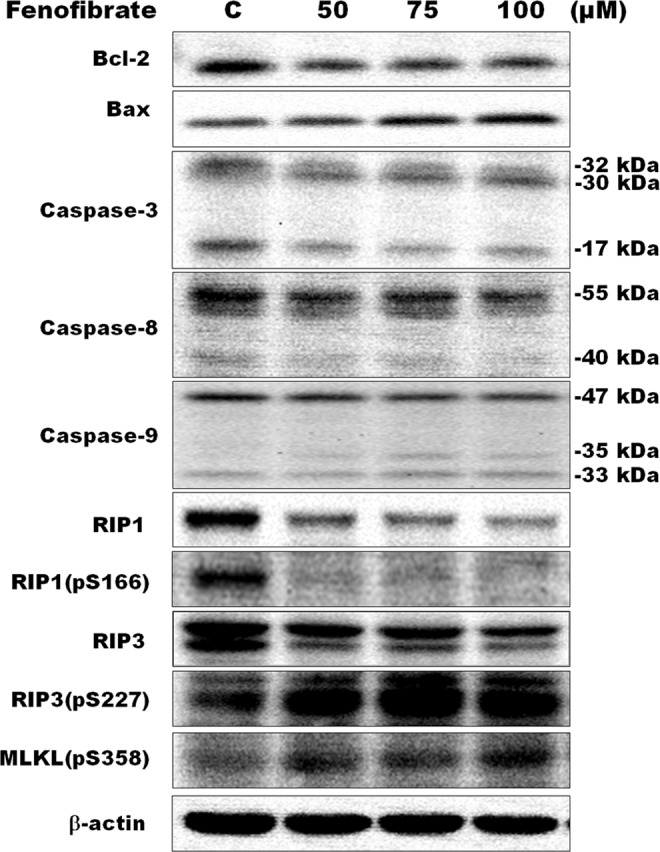
Effects of fenofibrate on the protein levels of apoptotic and necroptotic markers in Hep3B cells. Hep3B cells were incubated with 0.1% DMSO or with 50, 75 or 100 µM fenofibrate for 24 h. Cell lysates were analyzed by SDS-PAGE (8% for RIP1 and RIP1(pS166), 10% for caspase-8, MLKL(pS358), RIP3 and RIP3(pS227), 12% for caspase-9 and β-actin, 13% for Bcl-2 and caspase-3 and 14% for Bax), and then probed with primary antibodies followed by secondary antibodies. Results are representative of three independent experiments performed in triplicate. All of full-length gels and blots are included in Supplementary Fig. 6.

In this study, the annexin V/PI double staining was performed to demonstrate whether cell death induced by fenofibrate is linked to apoptosis or necrosis of Hep3B cells. Treatment of Hep3B cells with 50, 75 and 100 µM fenofibrate for 24 h resulted in a dose-dependent increase in total necrotic and late apoptotic rate in which a fenofibrate-induced dose-dependent increase of necrotic cells (Q1 area) was accompanied by a dose-dependent decrease in the late apoptotic cells (Q2 area) (Fig. 5A). Furthermore, treatment of Hep3B cells with 50 µM fenofibrate for 48 h exhibited a time-dependent increase in total necrotic and late apoptotic rate (Fig. 5A,B). No significant effect of the vehicle (DMSO) on total necrotic and apoptotic rate was observed (Fig. 5A,B). The nuclear morphology and DNA fragmentation of fenofibrate-sensitized Hep3B cells were also evaluated by DAPI staining and TUNEL assay. DAPI staining showed that treatment with 25, 50 and 100 μM fenofibrate for 24 h resulted in changes in nuclear morphology (Fig. 5C). A gradual increase in the number of cells with chromatin condensation, nuclear fragmentation and irregularly shaped nucleus were observed after 24 h of fenofibrate treatment (Fig. 5C). However, very few TUNEL-positive nuclei were observed in Hep3B cells incubated with fenofibrate for 24 h (Fig. 5C). TUNEL assay is a method for detecting DNA fragments. To obtain further support for the apoptosis induced by fenofibrate in Hep3B cells, the protein expression of the markers available to characterize apoptotic cell death, such as Bcl-2, Bax, caspase-3, caspase-8 and caspase-9 protein, were performed by Western blotting analysis. A decrease in Bcl-2 expression and an increase in Bax expression were observed in Hep3B cells after treatment with fenofibrate for 24 h (Fig. 6). Incubation with fenofibrate significantly decreased the protein levels of proform of caspase-3, -8 and -9 after treatment with 50, 75 and 100 µM fenofibrate for 24 h, while there were significant increases in the amount of the fragment of 30 kDa of caspase-3 and 35 kDa of caspase-9 protein levels in this study (Fig. 6). It is noteworthy that the decreases in the amount of the active form of 17 kDa of caspase-3 and 40 kDa of caspase-8 protein levels were observed after fenofibrate treatment (Fig. 6). This study has also demonstrated that fenofibrate-treated Hep3B cells revealed decreases in the relative abundance of RIP1, RIP1(pS166) and RIP3 proteins, while fenofibrate induced an increase in RIP3(pS227) protein levels after treatment with fenofibrate for 24 h (Fig. 6). RIP1 and RIP3 are well-known key signaling molecules in necroptosis. Furthermore, a fenofibrate-induced increase in MLKL(pS358) (mixed lineage kinase domain like protein) protein levels was observed in this study (Fig. 6). Based on the above data, we indicated that both apoptosis and necroptosis pathway might be involved in the fenofibrate-induced Hep3B cell death.

### The effects of fenofibrate on the expression of lipid metabolism-related proteins in Hep3B cells

**Figure 7:**
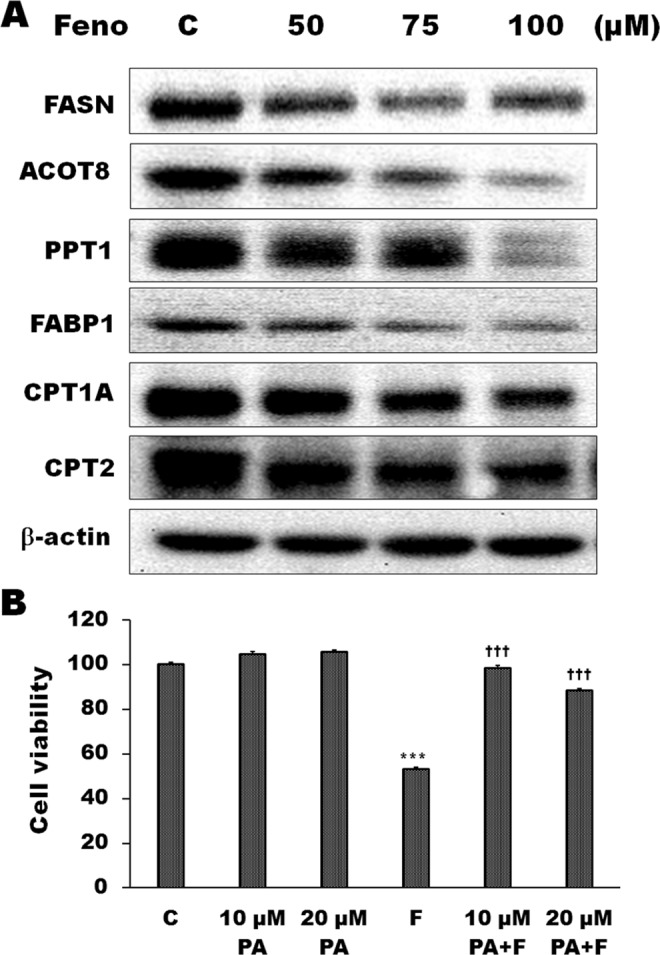
Effects of fenofibrate on the expression of lipid metabolism-related proteins in Hep3B cells. (**A**) The effects of fenofibrate on the protein levels of lipid metabolism-related proteins were detected by Western blot analysis. Hep3B cells were incubated with 0.1% DMSO or with 50, 75 or 100 µM fenofibrate (Feno) for 24 h. Cell lysates were analyzed by SDS-PAGE (5% for FASN, 7% for CPT1A, 10% for CPT2, 11% for ACOT8, 12% for β-actin, 13% for PPT1 and 15% for FABP1), and then probed with primary antibodies followed by secondary antibodies. Results are representative of three independent experiments performed in triplicate. All of full-length gels and blots are included in Supplementary Fig. 7. (**B**) Effects of palmitate on fenofibrate-induced Hep3B cell death. Cells were pretreated with 10 or 20 µM palmitate (PA) for 1 h and then 50 µM fenofibrate (F) for 24 h. The cytotoxicity was assessed by Trypan blue exclusion assay and the viable cells were counted. The data are expressed as the mean percentage of control ± S.D. of three independent experiments performed in duplicate. ^***^P < 0.001 compared to the control values. ^†††^*P* < 0.001 compared to the fenofibrate alone.

Alteration of lipid metabolism has been known to be involved in the development of cancers. The present study examined the effects of fenofibrate on the expression of lipid metabolism-related proteins, such as proteins containing thioesterase domain and fatty acid transport proteins, in Hep3B cells. As shown in Fig. 7A, fenofibrate induced the decrease of FASN protein expression during fenofibrate-induced Hep3B cell death by Western blotting techniques. This result is consistent with the result of molecular docking in which fenofibrate was found to be an inhibitor of FASN through binding on the thioesterase domain of FASN. In addition to FASN containing thioesterase domain, acyl-CoA thioesterase (ACOT) and palmitoyl protein thioesterase (PPT) are also thisoesterases associated with fatty acid metabolism. The protein levels of ACOT8 and PPT1 were decreased during treatment with 50, 75 and 100 μM fenofibrate for 24 h (Fig. 7A). Fatty acid-binding protein 1 (FABP1) plays a role in fatty acid uptake, transport and metabolism. The protein levels of FABP1 were decreased during treatment with 50, 75 and 100 μM fenofibrate for 24 h in Hep3B cells (Fig. 7A). The protein levels of carnitine palmitoyltransferase (CPT) were also examined. CPT1 and CPT2 act to transport long-chain fatty acid across the outer and inner mitochondrial membrane, respectively. Exposure of Hep3B cells to fenofibrate for 24 h resulted in significant decreases in CPT1 and CPT2 protein levels (Fig. 7A). We further investigated whether the cytotoxic effect of fenofibrate was induced by depletion of palmitate which is the final product catalyzed by FASN. Exogenous palmitate reversed the growth inhibition induced by fenofibrate in Hep3B cells (Fig. 7B). These results indicated that the inhibition of FASN reaction and lipid metabolism-related proteins expression could be an important pathway in fenofibrate-mediated cytotoxic effects in Hep3B cells.

## Discussion

From the result of docking simulation, fenofibrate is similar to orlistat, a FASN inhibitor, with respect to its ability to bind excellently to the thioesterase domain of FASN binding site. We hypothesized that fenofibrate could be a potential drug candidate for the cancer treatment and a new treatment option for cancers. The present study demonstrated that treatment of fenofibrate induced a significant cytotoxicity of human hepatoma Hep3B cells in a time-dependent manner. It is worthy to note that fenofibrate selectively causes cytotoxicity in different cancer cell lines. The present study demonstrated that Hep3B cells are significantly more sensitive to cell killing by fenofibrate than lung cancer cell line CH27 and oral cancer cell line HSC-3 cells. Furthermore, fenofibrate had no significant cytotoxic effect on the HepG2 cells, another hepatocellular carcinoma cell line. Slany *et al*. (2010) indicated that Hep3B cells are more closely related to fibroblasts, while HepG2 to hepatocyte^27^. Hep3B is derived from more differentiated liver cells in hepatic lobule and HepG2 is from neonatal hepatic progenitor cells with great potential^28^. Based on the above reasons, Hep3B and HepG2 come from different origins of biopsy specimens and different stages of hepatocyte differentiation that may support the differences in fenofibrate responses between Hep3B and HepG2 cells.

Bcl-2 and caspase-3 family members are crucial for the regulation of apoptosis. In this study, a reduction in Bcl-2 expression and an increase in Bax expression were observed in Hep3B cells after treatment with fenofibrate. This study also demonstrated that the decreases in protein levels of proform caspase-3, -8 and -9 are involved in fenofibrate-induced cell death of Hep3B cells. There are three bands, 32, 30 and 17 kDa, were observed on immunoblots of caspase-3 proteins. In general, caspase-3 gets activated by proteolytic cleavage into the 17 and 12 kDa active subunits during apoptosis. However, caspase-3 stimulated by fenofibrate was processed to yield two cleavages of 30 and 17 kDa in this study. The amount of 32 and 17 kDa of caspase-3 significantly decreased after treatment with fenofibrate, while an increase in the protein level of 30 kDa of caspase-3 was observed. Kuida *et al*. (1996) had indicated that a 32 kDa band corresponds to the proform of caspase-3 and a 30 kDa band represents the cleaved caspase-3 without its prodomain^29^. There are two typic apoptosis pathways, the extrinsic pathway involving caspase-8 in the receptor (Fas)-mediated apoptosis and the intrinsic mitochondrial pathway involving caspase-9. It is noteworthy that the decrease in the active form of 40 kDa of caspase-8 protein levels was observed after fenofibrate treatment in this study. However, the active forms of 35 kDa of caspase-9 protein levels were significantly increased after fenofibrate treatment. Our results are in agreement with earlier researches that intrinsic pathway involvement in apoptosis, as evidenced by changing the expression of caspase family and Bcl-2 family members, has been found in several tumor cell lines following pharmacological FASN inhibition^30–32^.

Besides apoptosis induction, exposing the Hep3B cells to fenofibrate also induced an increase in the necrotic cells according to the result of the experiment of annexin V/PI double staining in this study. Recent advances have defined a caspase-independent regulated form of cell death as necroptosis, a highly regulated process that occurs when caspase-8 is inhibited^33,34^. In the absence of active caspase-8, receptor-interacting protein kinases 1 (RIP1) and RIP3 autophosphorylate and transphosphorylate each other, leading to the formation of necrosome to initiate necroptosis^35^. Therefore, many reports have suggested that RIP1 and RIP3 kinases are important regulators in necroptosis^36,37^. The present study demonstrated that fenofibrate-treated Hep3B cells revealed increases in the relative abundance of RIP3(pS227) after treatment with fenofibrate for 24 h. However, the fenofibrate-induced decrease in RIP1, RIP1(pS166) and RIP3 protein levels was observed. It is interesting to note that fenofibrate did not stimulate RIP1 phosphorylation which was demonstrated that RIP1 was phosphorylated by RIP3. Recently, it has been indicated that RIP1 may not be considered necessary to induce necroptosis. Overexpression of active RIP3 has also been demonstrated to induce necroptosis regardless of the existence of RIP1, suggesting RIP3 seems to participate solely in necroptosis^38–40^. Furthermore, RIP1 has been recognized to be involved in both apoptosis and necroptosis in certain cell lines^41,42^. In this study, fenofibrate triggers both apoptosis and necroptosis, therefore, the complication of the role of RIP1 and RIP3 is imaginable in fenofibrate-induced Hep3B cells death.

The MLKL was demonstrated to be an important downstream target of RIP3^43,44^. Phosphorylation of RIP3 on Ser227 leads to MLKL phosphorylation at the threonine 357 and serine 358 residues, and subsequently induced necroptosis^44–46^. Furthermore, it has been indicated that blocking MLKL activity is critical for the execution of necroptosis inhibition^44^. In this study, the fenofibrate-induced increase in MLKL(pS358) protein levels was observed. In addition, necroptotic cell death and the highest level of phosphorylated RIP3, critical signaling molecule for necroptosis, were observed after Hep3B cells treatment with fenofibrate for 24 h. Although the entire molecular mechanism of fenofibrate-induced Hep3B cells necroptosis is unclear, RIP3/MLKL might be a mediator of necroptosis signaling in this study.

Alteration of lipid metabolism has been known to be involved in the development of cancer. The present study demonstrated that fenofibrate, an effective lipid-lowering drug, had a significant effect on the expression of lipid metabolism-related proteins, such as protein containing thioesterase domain and fatty acid transport proteins, in Hep3B cells. The protein levels of FASN, ACOT8 and PPT1, protein containing thioesterase domain, were significantly decreased during treatment with fenofibrate for 24 h in Hep3B cells. Hung *et al*. (2014) have indicated that ACOT8 is frequently overexpressed in hepatocellular carcinoma specimens and blocking ACOT8 may inhibit hepatocellular carcinoma cell proliferation *in vitro*^47^. In addition, we also demonstrated that fenofibrate induced a reduction of the protein levels of liver fatty acid-binding protein (FABP1). FABP1 is a regulator in fatty acid metabolism, transport and uptake and plays a key role in hepatic lipid metabolism^48,49^. Since fatty acid oxidation is the major process of energy production in cells, many researches confirmed the link between mitochondrial β-oxidation of fatty acid and the proliferation and apoptosis of cancer cells^50,51^. CPT1 and CPT2, which are important for transport long-chain fatty acid across the outer and inner mitochondrial membrane, are critical in mitochondrial β-oxidation of fatty acid. In this study, there was a significant decrease in protein expression pattern of CPT1 and CPT2 compared to control cells. These results suggested that fenofibrate might induce the decrease of energy generation or reduction of ATP supply and impairement cell proliferation by inhibition of the protein expression of CPT1 and CPT2, resulting in Hep3B cell death. Based on the above data, the decrease in fatty acid uptake and transport is involved in fenofibrate-induced Hep3B cell death. Experiments with exogenous palmitate indicated that the FASN inhibition by fenofibrate blocked synthesis of palmitate, the enzymatic ultimate product of fatty acid synthase, which caused an alteration of lipid synthesis and then induced Hep3B cell death in this study. Many recent studies have confirmed that palmitate and lipid synthesis were associated with energy metabolism and membrane building in tumor cells^52–54^.

In this study, fenofibrate induced cell cycle S and G2/M arrest, which was accompanied by a marked reduction in the G1 in Hep3B cells. In general, cyclin D1 and E are important regulators in the G1 phase and in the G1-S phase transition, respectively. In this study, the protein expression of cyclin D1 and E was significantly decreased by fenofibrate. It is well-known that Cdk2 activity is limited on the G1 and S phase, and is required for the transition of the cell cycle into S phase. The binding of Cdk2 with cyclin E is essential for the G1/S transition, and binding to cyclin A is necessary for S phase progression^55^. As expected, fenofibrate induced a significant increase in the cyclin A protein in Hep3B cells in this study. It is noteworthy that two bands of Cdk2 appear at approximately 33 kDa. The signal of lower band of Cdk2 was found to gradually increase up to 100 μM of fenofibrate, whereas the upper band decrease. In our previous study, the protein expression of Cdk2 also displays two bands at about 33 kDa. 4β-Hydroxywithanolide E induced a marked increase in the amount of upper band and a significant reduction in the amount of lower band in MCF-7 cells^56^. A G1-phase arrest of the cell cycle was observed when MCF-7 cells were incubated with 4β-hydroxywithanolide E for 24 h in our previous study^56^, whereas fenofibrate induced cell cycle S and G2/M arrest in this study. Therefore, we indicated that the protein expression of lower band of Cdk2 might play a key role in regulation of the transition from G1 to S phase and S phase progression. Jeffrey *et al*. (1995) have demonstrated that cyclin A binding with Cdk2 is required for progressing through the S phase while association with Cdk1 is required for entry into M phase^55^. In this study, fenofibrate also induced a marked increase in the protein levels of Cdk1 compared to the control cells. P21 (CIP1/WAF1), a potent Cdk inhibitor, is primarily associated with inhibiting the kinase activity of Cdk2-cyclin E/cyclin A complex which plays a critical role in the regulation of the G1/S transition of cell cycle^57,58^. Gottifredi *et al*. (2004) indicated that the complex of cyclin A and Cdk2 cannot entirely be saturated by a small amount of p21 detected during S phase arrest and decreased p21 levels could be necessary for effective restart DNA synthesis^59^. Furthermore, Gottifredi *et al*. (2004) also suggested that proteasome-mediated p21 turnover induces a significant decrease in p21 protein expression when cells are arrested in S phase^59^. In this study, fenofibrate caused a significant decrease in p21 protein expression accompanied by the increase in cyclin A and cyclin E protein expression, thereby promoting G1/S transition and S phase progression of cells.

Based on the above data, we demonstrated that inhibition of cell proliferation by fenofibrate was accompanied by inhibiting the expression of key enzymes in fatty acid metabolisms. Treatment of Hep3B cells with fenofibrate resulted in cell growth inhibition and induction of S and G2/M cell cycle arrest with a significant change in the protein expression of S- and G2/M-phase regulators. Fenofibrate-induced Hep3B cells apoptosis and necroptosis were demonstrated to be associated with the changes of expression of apoptotic and necroptotic markers. Inhibition of lipid metabolism-related proteins expression was involved in the effects of fenofibrate on cell death of Hep3B cells. These results suggest that fenofibrate has an anti-cancer effect in Hep3B cells and inhibition of lipid metabolism may be involved in fenofibrate-induced Hep3B cells apoptosis and necroptosis.

## Materials and Methods

### Materials

Fenofibrate, palmitic acid, propidium iodide and RNase A were purchased from Sigma Chemical Company (St. Louis, MO, USA). Annexin V-FITC apoptosis detection kit was purchased from BioVision (Mountain view, CA, USA). Antibodies to various proteins were obtained from the following sources: β-Actin was from Sigma Chemical Company. Bax, caspase-3, Cdk1, Cdk2, Cdk4, cyclin B and p21 were purchased from BD Biosciences (San Diego, CA, USA). Cyclin D1, MLKL(pS358) and RIP3(pS227) were purchased from Abcam (Cambridge, MA, USA). ACOT8, Bcl-2, caspase-8, CPT1A, CPT2, cyclin A, cyclin E, FABP1, FASN, PPT1, RIP1 and RIP3 were from GeneTex Inc (Irvine, CA, USA). Caspase-9 and RIP1(pS166) were purchased from Cell Signaling Technology Inc (Danvers, MA, USA). Horseradish peroxidase (HRP)-conjugated goat anti-rabbit and -mouse IgG were from Abcam.

### Molecular docking

In order to predict the inhibitory activity of fenofibrate on FASN, the computational simulation of fenofibrate with thioesterase of FASN was performed. The 2.3 Å resolution crystal structure of thioesterase-orlistat complex (2px6) recovered from the RCSB Protein Data Bank (http://www.rcsb.org/pdb) was used as the target for molecular docking. The docking calculations of fenofibrate with thioesterase domain were performed with LigandFit program within the software package Discovery Studio 3.0 (Accelrys, San Diego, CA, USA). Protein structure of 2px6 was prepared as described previously^60^.

### Cell culture

Human hepatocellular carcinoma cell lines Hep3B and HepG2, oral cancer cell line HSC-3 and lung cancer cell line CH27 were cultured in Dulbecco’s modified Eagle’s medium (Life Technologies, Rockville, MD, USA) supplemented with 10% fetal bovine serum (HyClone, Logan, UT, USA), 100 U/ml penicillin, 100 µg/ml streptomycin and 2 mM glutamine at 37 °C in a humidified atmosphere with 5% CO_2_. Human hepatoma Hep3B and HepG2 cells were purchased from the Food Industry Research and Development Institute (Hsinchu, Taiwan). Hep3B cells are more closely related to fibroblasts with EMT (epithelial to mesenchymal transition), while HepG2 to hepatocyte^28^. The human oral cancer cell line HSC-3 was kindly provided by Professor Jing-Gung Chung (China Medical University, Taichung, Taiwan). The human lung cancer cell line CH27 was kindly provided by Professor Shih-Lan Hsu (Taichung Veterans General Hospital, Taichung, Taiwan).

### Cytotoxicity assay

Cell viability was measured by Trypan blue exclusion assay as described previously^60^.

### Cell cycle analysis

Cell cycle analysis was performed as described previously^61^. Fixed cells were incubated with propidium iodide (50 µg/ml) and analyzed using a FACScan flow cytometer (Becton Dickinson Instruments).

### Protein preparation and Western blot analysis

Protein preparation and Western blot analysis were performed as previously described^61^. The proteins (50 µg) were separated by SDS-PAGE, and then electrotransferred onto polyvinylidene fluoride membranes (Millipore, Bedford, MA, USA). Membranes were probed with primary antibodies followed by secondary antibodies with horseradish peroxidase. The primary antibodies used in this study were as follows. β-Actin, 1:5000; ACOT8, 1:500; Bax, 1:2000; Bcl-2, 1:1000; caspase-3, 1:1000; caspase-8, 1:5000; caspase-9, 1:200; Cdk1, 1:2500; Cdk2, 1:2500; Cdk4, 1:500; CPT1A, 1:500; CPT2, 1:500; cyclin A, 1:500; cyclin B, 1:500; cyclin D1, 1:500; cyclin E, 1:500; FABP1, 1:1000; FASN, 1:1000; MLKL(pS358), 1:1000; p21, 1:500; PPT1, 1:500; RIP1, 1:1000; RIP1(pS166), 1:1000; RIP3, 1:500; RIP3(pS227), 1:500. β-Actin was used as an internal control.

### Annexin V-FITC/PI double staining assay

After treatments, annexin V-FITC/PI double staining assay were performed as previously described^61^.

### Nuclear staining and terminal deoxynucleotidyl transferase dUTP nick end labeling (TUNEL) assay

After treatment, cells were fixed with 3.7% formaldehyde for 20 min and permeabilized with 0.1% Triton X-100 for 10 min. After permeabilization, cells were stained with TUNEL and then stained with 1 μg/ml DAPI (4′,6′-diamidino-2-phenylindole dihydrochloride) for 5 min at 37 °C. After three washings in PBS, the cells were observed by fluorescence microscopy. TUNEL assay was performed following the manufacturer’s instructions (Roche Applied Science, Indianapolis, IN, USA). The instructions indicated that false negative results could be obtained because DNA cleavage can be absent or incomplete in some forms of apoptotic cell death^62^.

### Data analysis and statistics

Statistically significant difference between groups was identified by Student’s two-tailed *t*-test. *P* < 0.05 was considered to be significant.

## Supplementary information

full-length gels and blots
