# Supplementary material for: Fenofibrate induces human hepatoma Hep3B cells apoptosis and necroptosis through inhibition of thioesterase domain of fatty acid synthase
Source: Sci Rep. 2019 Mar 1;9:3306. doi: 10.1038/s41598-019-39778-y (PMC6397239; doi:10.1038/s41598-019-39778-y)

Fenofibrate induces human hepatoma Hep3B cells apoptosis and necroptosis through inhibition of thioesterase domain of fatty acid synthase

Bang-Jau You[1,†], Mann-Jen Hour[2,†], Li-Yun Chen[2], Shu-Ching Luo[2], Po-Hsiang Hsu[2] & Hong-Zin Lee[2,*]

Initial pictures of Western blot in this article.

Omics Bio Prestained Protein Marker (Tris-Glycine 4-20%) was used in this study.

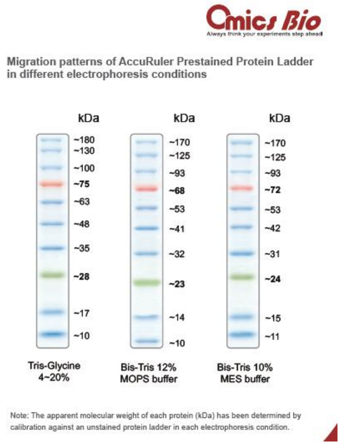

Figure 4

Cdk1

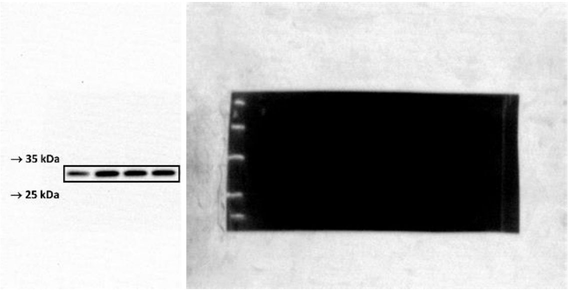

Cdk2

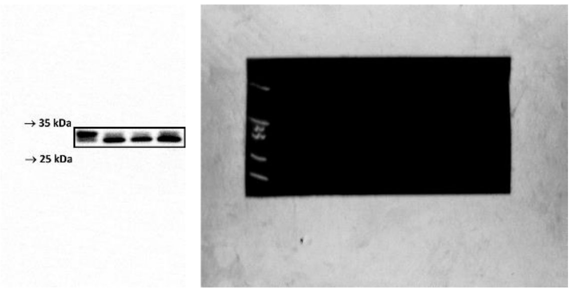

Cdk4

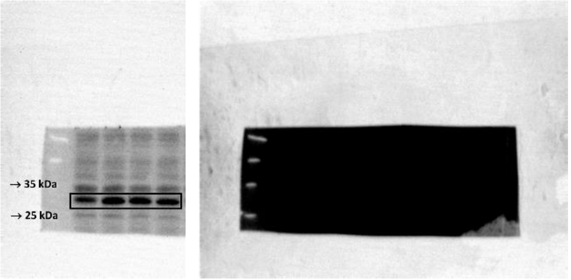

cyclin A

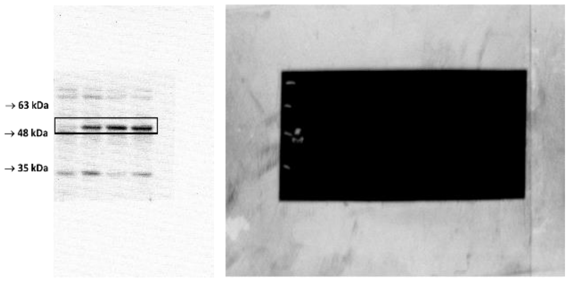

cyclin B

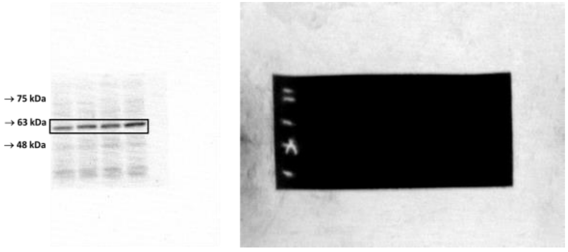

cyclin D1

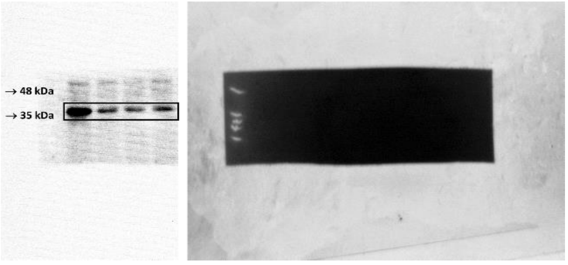

cyclin E

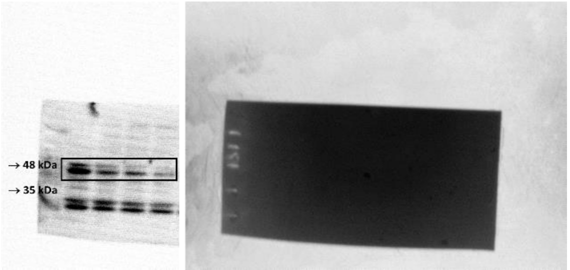

p21

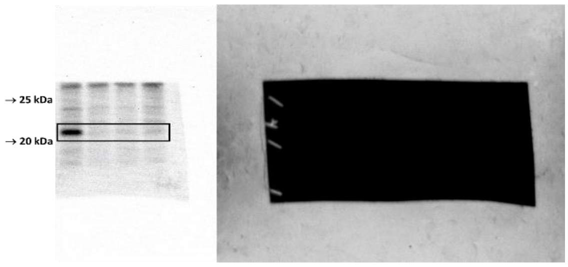

β-actin

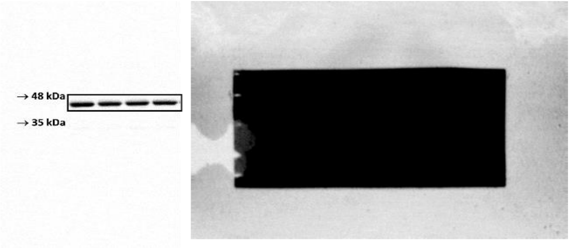

Figure 6

Bcl-2

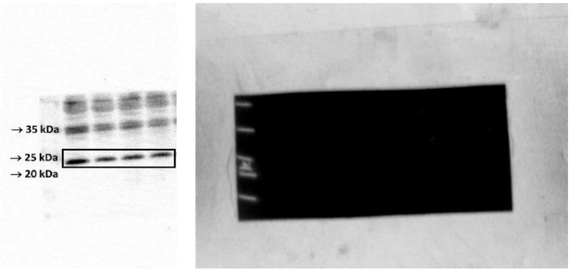

Bax

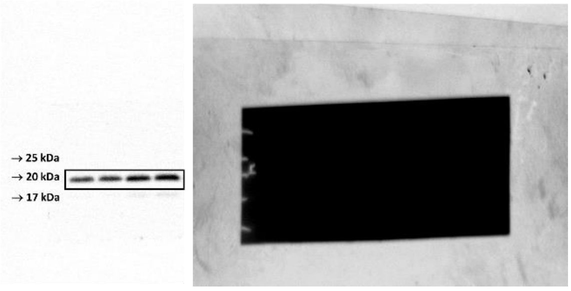

Caspase-3

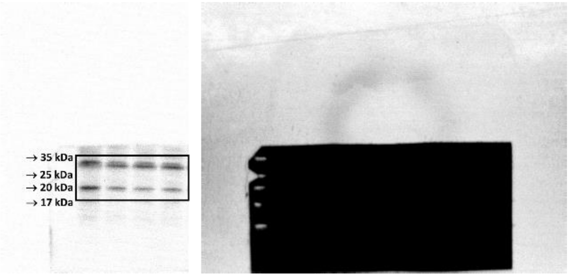

Caspase-8

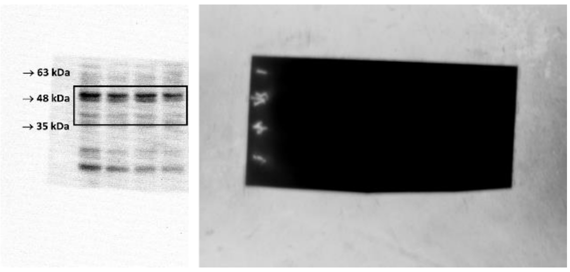

Caspase-9

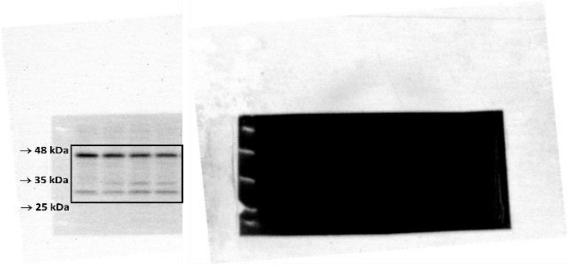

RIP1

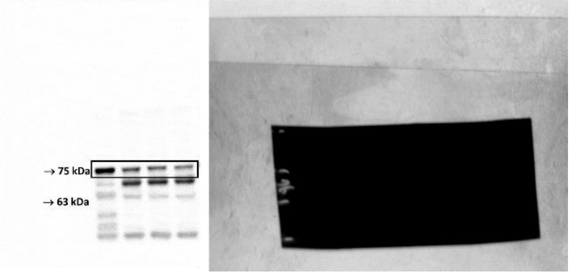

RIP1(pS166)

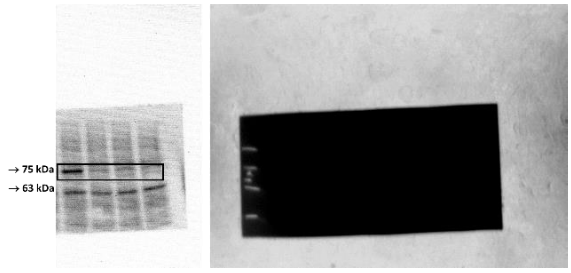

RIP3

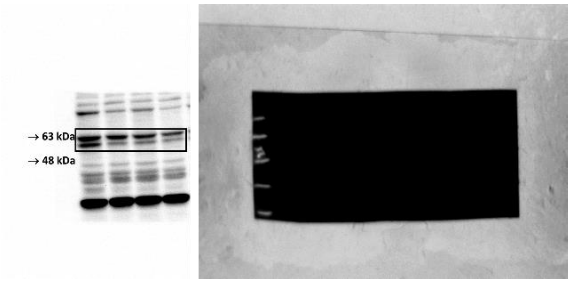

RIP3(pS227)

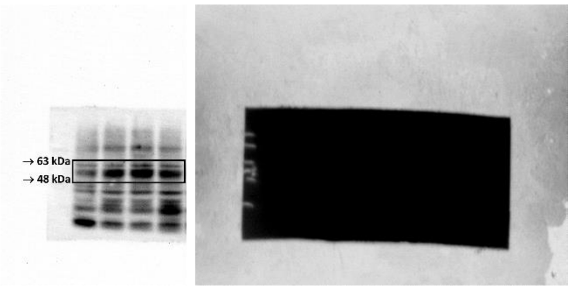

MLKL(pS358)

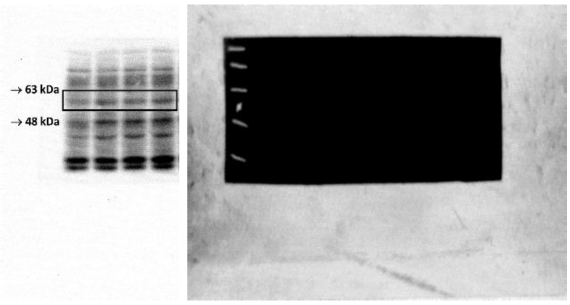

β-actin

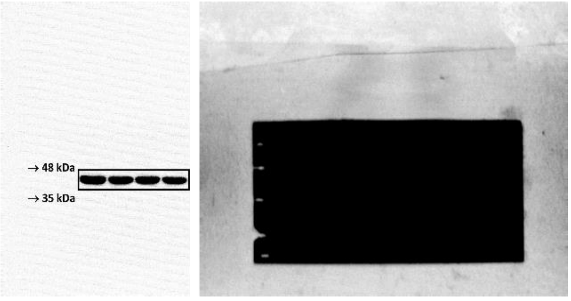

Figure 7

FASN

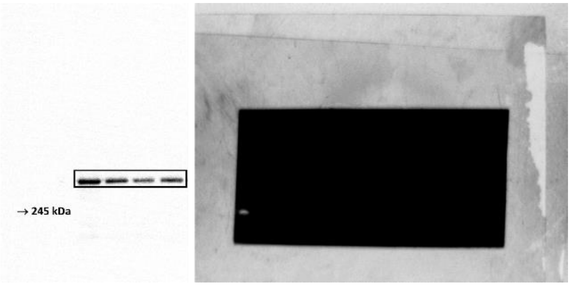

ACOT8

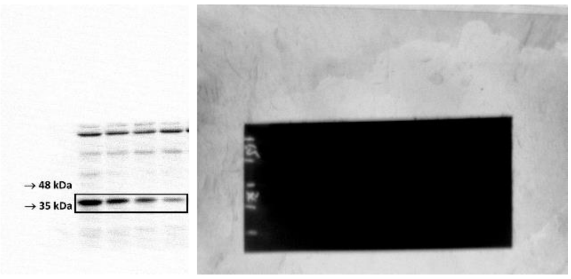

PPT1

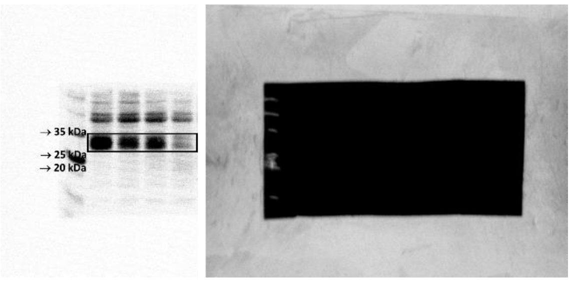

FABP1

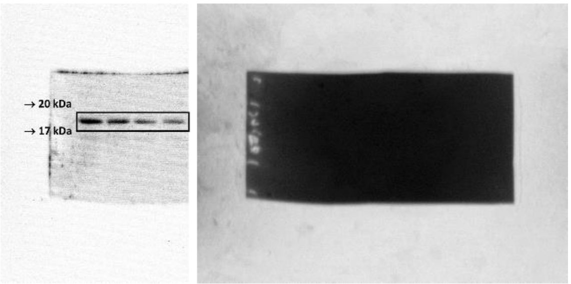

CPT1A

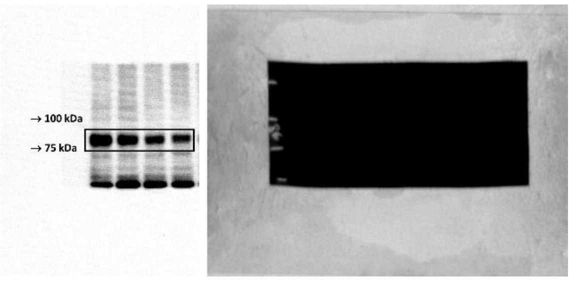

CPT2

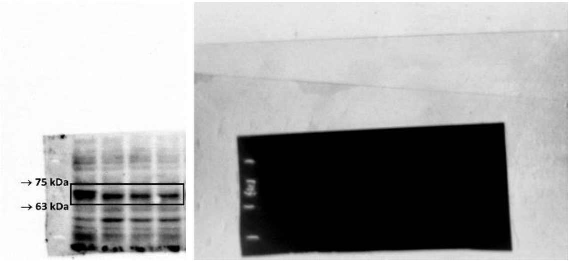

β-actin

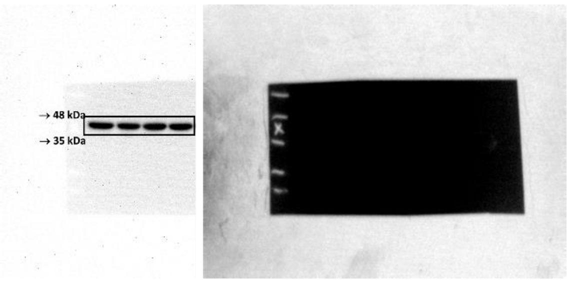

Supplement: Supplementary file 1 — full-length gels and blots [file 41598_2019_39778_MOESM1_ESM.pdf]
